# A Pilot Cross-Over Study to Evaluate Human Oral Bioavailability of BCM-95^®^CG (Biocurcumax™), A Novel Bioenhanced Preparation of Curcumin

**DOI:** 10.4103/0250-474X.44591

**Published:** 2008

**Authors:** B. Antony, B. Merina, V. S. Iyer, N. Judy, K. Lennertz, S. Joyal

**Affiliations:** Arjuna Natural Extracts Ltd., R&D Laboratory, P.B No. 126, Bank Road Alwaye-683 101, India; 1 Little Flower Hospital & Research Centre, Angamaly - 683 572, India; 2 Life Extension Foundation, Florida, USA

**Keywords:** Curcumin, bioavailability, biocurcumax, *Curcuma longa*, anticancer, antioxidant

## Abstract

Curcumin, the bioactive component of turmeric, *Curcuma longa* has an exceptionally wide spectrum of activities including antioxidant, anti-inflammatory and anti-cancer properties, and is currently under different phases of clinical trials for various types of soft tissue cancers. However, although *in vitro* and animal studies have shown anticancer activities of curcumin for virtually all types of human cancers, its poor bioavailability in the human body has severely limited its application to these diseases. Methods to increase its oral bioavailability are a subject of intense current research. Reconstituting curcumin with the non-curcuminoid components of turmeric has been found to increase the bioavailability substantially. In the present clinical study to determine the bioavailability of curcuminoids, a patented formulation, BCM-95^®^CG was tested on human volunteer group. Normal curcumin was used in the control group. Curcumin content in blood was estimated at periodical intervals. After a washout period of two weeks the control group and drug group were crossed over BCM-95^®^CG and curcumin, respectively. It was also compared with a combination of curcumin-lecithin-piperine which was earlier shown to provide enhanced bioavailability. The results of the study indicate that the relative bioavailability of BCM-95^®^CG (Biocurcumax™) was about 6.93-fold compared to normal curcumin and about 6.3-fold compared to curcumin-lecithin-piperine formula. BCM-95^®^CG thus, has potential for widespread application for various chronic diseases.

Curcumin, (diferuloylmethane; 1,7–bis[4-hydroxy-3-methoxyphenyl]–1,6-heptadiene-3,5-dione) along with its mono and di demethoxy derivatives, collectively called curcuminoids, constitute the major colouring matter and the biologically active constituents of *Curcuma longa* L. or turmeric. Ayurveda, Unani, Siddha and Chinese medicines recommend turmeric for a wide range of disorders and diseases. Modern science has provided a scientific basis for such uses 1–7. Curcumin has been shown to be a very powerful antioxidant more potent than tocopherols, a comprehensive antiinflammatory, and an anti cancer compound beneficial in virtually all forms of human cancers, including cancers refractory to common anticancer drugs. Curcumin has been shown to enhance the effects of common anti-cancer drugs, and doses as high as 8 to 12 g per day have been shown to be well tolerated in humans^8^,^9^. While all anticancer drugs weaken the immune system, curcumin enhances it and acts as an immunorestorer 10–19. Curcumin has been shown to be useful in a number of other chronic human ailments. For example, a human clinical trial is presently underway to test its efficacy in treating Alzheimer’s disease^20^. Curcumin is able to modulate several molecular targets, including transcription factors, cell cycle proteins, cytokines and chemokines, a multitude of enzymes, receptors and cell surface adhesion molecules^21^.

In comparison with many diet-derived polyphenols and anti-cancer drugs, the bioavailability of curcumin has been poor 22–34. For this reason, no clinical trial has progressed beyond the Phase I stage, and the wide range of physiological activities of curcumin has not yet been translated into clinical benefit. Poor absorption from the gut and avid metabolism in the body is cited as reasons for the lack of systemic availability. While the major portion of ingested curcumin is excreted through the feces unmetabolized, the small portion that is absorbed is extensively converted to its water-soluble metabolites, glucuronides and sulfate, and excreted. This seriously limits curcumin to reach targets distant from the gut and exert its beneficial action.

There have been sporadic attempts to increase the bioavailability of curcumin. Piperine, an inhibitor of UDP-glucuronosyltransferase, administered along with curcumin has been found to significantly enhance the plasma curcumin concentration in animals and in humans^35^. However, piperine is toxic in experimental animals^36^–^39^. Other formulations are currently under development^40^–^43^. While these developments are definitely encouraging, these require sophisticated technologies if and when they are fully developed. Such formulations are also unlikely to be cost-effective.

This study focused on a simple and cost-effective formulation, trademarked BCM-95^®^CG (Biocurcumax™), taking advantage of the synergism between the sesquiterpenoids present in turmeric and the curcuminoids^44^. Studies on experimental animals showed the efficacy of this formulation^45^. This communication presents the results on the relative bioavailability of normal curcumin and that of BCM-95^®^CG (Biocurcumax™) in human volunteers. The study also compares the relative bioavailability of BCM-95^®^CG (Biocurcumax™) with that of curcumin-lecithin-piperine formula.

## MATERIALS AND METHODS

BCM-95^®^CG (Biocurcumax™) 500 mg capsules were supplied by Arjuna Natural Extracts Ltd. Curcumin capsules (control) and curcumin-lecithin-piperine capsules were supplied by Life Extension, USA and the standard curcumin for quantitation was obtained from Sigma Chemical Co., USA. Chromatographic solvents and all other chemicals were purchased from Merck.

### Human subjects and study design:

This clinical trial was cleared by the Institutional Ethics Committee of the Little Flower Hospital, Angamali, Kerala, India on the request of the sponsor M/s Arjuna Natural Extracts Ltd, Kerala, India. Informed written consent from each of the prospective volunteers was obtained and after a medical examination those satisfying the inclusion and exclusion criteria, 11 subjects under the age group of 28–50 y were recruited for the study. The selected subjects were apparently healthy, and not diagnosed for any major diseases, as well as not presently taking any prescription medicines, and were not habitual users of tobacco and alcohol. The volunteers abstained from the consumption of aspirin or other NSAIDS and all foods containing turmeric two days prior to the study date.

### Administration of BCM-95^®^CG (Biocurcumax™), curcumin and curcumin-lecithin-piperine formula:

The volunteers were divided into three groups of 4, 4, and 3 and admitted to the hospital day before the trial. After overnight fasting, group1 volunteers (4 numbers) consumed 4×500 mg capsules of BCM-95^®^CG (Biocurcumax™) while group 2 (4 numbers) and group 3 (3 numbers) consumed control curcumin and equivalent doses of curcumin-lecithin-piperine formula, respectively. Blood was drawn from each volunteer just prior to dosing and at 1, 2, 3, 4.5, 6 and 8 h post-drug. After wash out period of two weeks the subjects crossed-over to the other drug, i.e, group 1 subjects were administered control curcumin and the group 2 and 3 were consumed BCM-95^®^CG (Biocurcumax™) capsules. The same protocol was followed.

### Extraction and quantitation of curcumin from plasma:

The blood (5 ml) was centrifuged at 2000×g for 10 min and the plasma was carefully drawn and collected in weighed tubes and the weights of plasma was recorded and were frozen till analyzed. Each sample of plasma (2.5 g) was allowed to attain room temperature and extracted with 3+2+2 ml ethyl acetate successively and the pooled extracts were filtered into evaporation tubes and the solvent evaporated to dryness under a stream of nitrogen at 40–45^°^ in a Turbo Vap Concentration Work Station (Caliper Life Sciences, USA). The dried samples were dissolved in 2 ml of methanol (HPLC grade) using a vortex mixer. It is then analyzed by HPLC in a Shimadzu LC 10AT Liquid Chromatograph System with SPD-10 A UV detector in isocratic mode. The column used was C_18_ ODS Phenomenex (250×4.6 mm, 5 μ particle size) with methanol as the mobile phase and the detection wave length 420 nm. Peaks were assigned in comparison with standard curcumin (Sigma). Curcumin was quantitated by using a calibration graph obtained from standard curcumin.

## RESULTS AND DISCUSSION

**Fig. 1:**
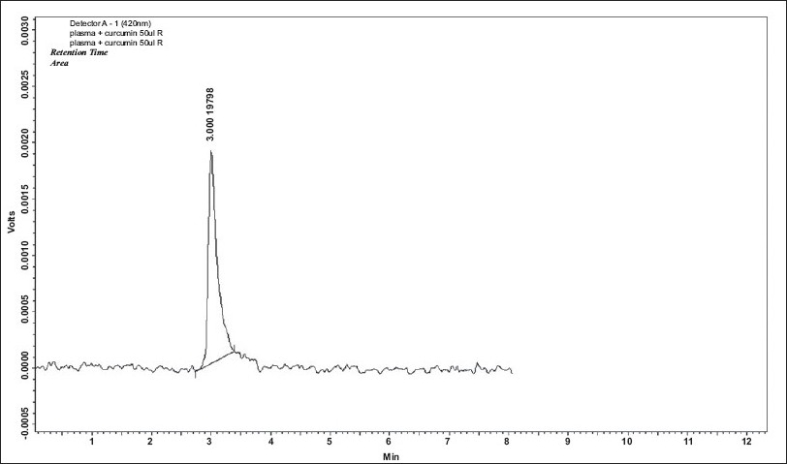
HPLC profile of curcumin from blood plasma. Blood plasma was extracted with ethyl acetate successively; all the washings were pooled and dried. This was dissolved in methanol and 20μl of this solution was injected into the HPLC column. The retention time obtained was 3 min which is the same for standard curcumin.

**Fig. 2:**
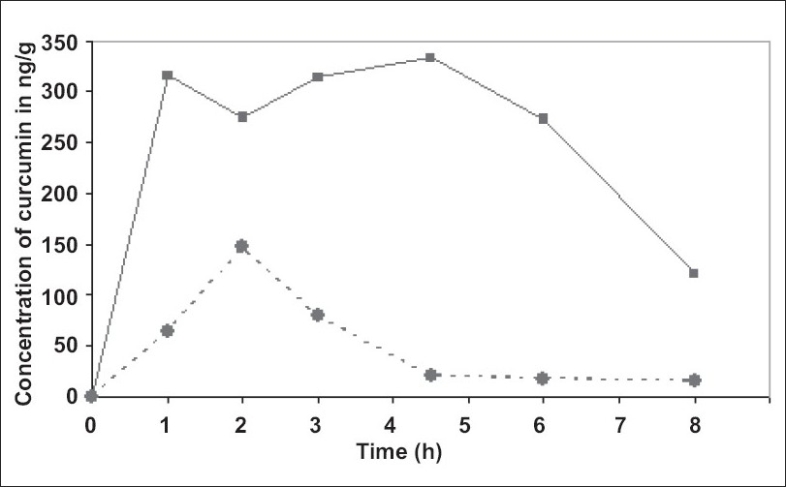
Mean plasma concentration of curcumin from BCM-95^®^CG (Biocurcumax™) and curcumin control. This figure shows the bioavailability of curcumin from blood plasma of the subjects taking BCM-95^®^ CG (Biocurcumax™) and curcumin at a single dose of 2000 mg. Blood was drawn before consuming the capsules (0 h) and 1, 2, 3, 4 ½ 6 and 8 h. (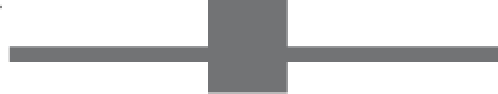) shows the concentration of curcumin in the BCM-95^®^ CG (Biocurcumax™) group whereas (
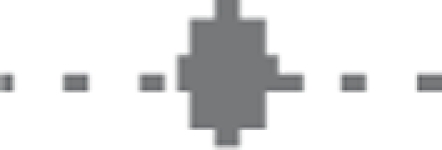
) that of curcumin group.

**Fig. 3:**
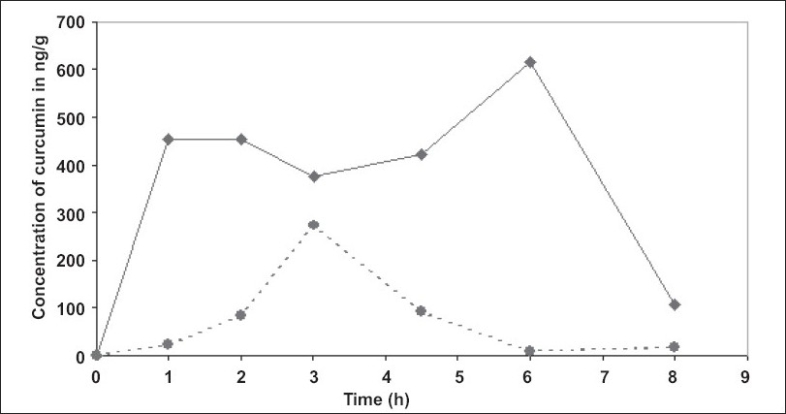
Mean plasma concentration of curcumin from BCM-95^®^CG (Biocurcumax™) and curcumin-lecithin-piperine groups. This figure shows the bioavailability of curcumin from blood plasma of the subjects taken BCM-95^®^ CG (Biocurcumax™) and curcumin-lecithin-piperine complex at a single dose of 2000 mg. The blood was drawn before consuming the capsules (0 h) and 1, 2, 3, 4 ½,, 6 and 8 h (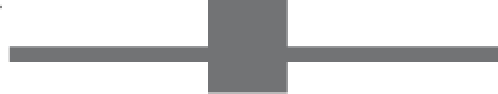) shows the concentration of curcumin in the BCM 95^®^ CG (BiocurcumaxTM) group where as (
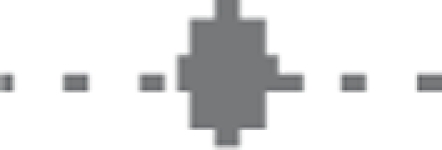
) that of curcumin + lecithin + piperine complex group.

There is an urgent need to find ways and means to enhance the bioabsorbability of curcumin so that its potential beneficial effects can be realized clinically. Results of the present study indicate that the proprietary formulation BCM-95^®^CG (Biocurcumax™) largely meets such a demand. The product at 2 g/day levels was well tolerated by all the volunteers who had participated in the study without even mild adverse reactions. The method of estimation of curcumin from blood plasma was validated by doing the recovery (80%) analysis. The peaks obtained for the curcumin reference standard as well as curcumin from the blood plasma were matched and typical plasma sample profile is shown in fig. 1. The concentration time profile of control curcumin versus BCM-95^®^CG (Biocurcumax™) is shown in fig. 2 and that of curcumin-lecithin-piperine versus BCM-95^®^CG (Biocurcumax™) is shown in fig. 3. The values were analysed statistically by Anova and the difference shown by test and control groups were significant (*P* < 0.05). As evident from fig. 2 the absorption of curcumin was faster from BCM-95^®^CG (Biocurcumax™) peaking in the first hour (mean 315.8 ng/g). This value dropped a bit during the succeeding hour at 274.6 ng/g and then reached the maximum at 4.5 h (456.88 ng/g). Thereafter the values gradually decreased. However, even at 8 h some residual curcumin remained in the blood. In contrast, the absorption of control curcumin was relatively slower, peaking at 2 h (149.8 ng/g) and then virtually disappeared from the blood by 4.5 h. Considering that one hour is too short a time period for the ingested curcumin to reach the intestine and then into blood, it may be speculated that at least part of the curcumin from BCM-95^®^CG (Biocurcumax™) is absorbed extra-intestinally. Apparently, this does not happen with ordinary curcumin.

BCM-95^®^CG (Biocurcumax™) may also probably modulate the Phase II enzymes due to its longer presence in the tissues and blood. Curcumin is simultaneously a substrate for these enzymes, an inducer of this enzymes as well as an inhibitor of these enzymes, depending on the context. However, which pathway is operating in individual cases is difficult to predict. Further, owing to its longer presence, curcumin from BCM-95^®^CG (Biocurcumax™) may modulate also the activity of the efflux pump, P-glycoprotein. All these activities may help to improve the absorption of curcumin from the present formulation compared to normal curcumin.

The pharmacokinetic data were arrived at using WinNonlin software (Table 1). BCM-95^®^CG (Biocurcumax™) showed a mean t 1/2 (half-life) of 4.96 h, Ke (elimination rate constant) of 0.26 h^-1^while the corresponding values for curcumin were 2.6 h and 0.296 h^-1^, respectively. The area under the curve (AUC) of BCM-95^®^CG (Biocurcumax™) was about 6.93 times that of curcumin control meaning a bioavailability enhancement of 693% (Biocurcumax™ (AUC)–3201.28, Curcumin (AUC)-461.86). The bioavailability of BCM-95^®^CG (Biocurcumax™) versus curcumin-lecithin-piperine formula, as seen in fig. 3, also shows an enhancement of 6.37. BCM-95^®^CG (Biocurcumax™) had better bioavailability than curcumin-lecithin-piperine formula, and well as a longer retention time compared to the latter.

**TABLE 1:** AVERAGE VALUE OF PHARMACOKINETIC PARAMETERS OF CURCUMIN CONTROL AND BCM-95^®^CG (BIOCURCUMAX™)

Parameter	Curcumin	BCM-95^®^CG (Biocurcumax™)
Tmax	2	3.44
Cmax	149.8	456.88
Ke	0.296	0.26
t 1/2	2.63	4.96
AUC (0.inf)	461.86	3201.28
Cl (observed)/F	0.006735	0.001682
Vz (observed)/F	0.026362	0.006784

This table shows the average value of pharmacokinetic parameters of curcumin control and BCM-95^®^ CG (Biocurcumax™). Tmax: Time of Peak plasma concentration, Cmax: Peak plasma concentration, Ke: Elimination rate constant, t1/2: Half life, AUC(0-infinity): Area under curve from ‘0’ h to infinity, Cl/F: Clearance/Bioavailability, Vd/F: Volume of distribution/bioavailability

The mean plasma concentration of curcumin from curcumin-lecithin-piperine formula reached a peak value of 344.32 ng/g and a Tmax of 3.5 h while the corresponding values for curcumin from BCM-95^®^CG (Biocurcumax™) were 689.18 ng/g and 4.67 h, respectively (Table 2). The elimination rate constant (Ke) for curcumin-lecithin-piperine formula was 0.3372 h^-1^compared to 0.139 h^-1^ of BCM-95^®^CG (Biocurcumax™), thus clearly establishing the difference between BCM-95^®^CG (Biocurcumax™) and curcumin-lecithin-piperine formula. The area under the curve (AUC) 0 to infinity for curcumin-lecithin-piperine formula was 624.26 units while that of BCM-95^®^CG (Biocurcumax™) was 3975 units. Thus, BCM-95^®^CG (Biocurcumax™) has a relative bioavailability of 6.37 compared to that of curcumin-lecithin-piperine formula. BCM-95^®^CG (Biocurcumax™) should also have a better safety profile because the composition is made entirely from turmeric in sharp contrast to the formulation containing piperine which is toxic to experimental animals^36^–^39^. In our hands, this composition improved the bioavailability of curcumin only marginally.

**TABLE 2:** AVERAGE VALUE OF PHARMACOKINETIC PARAMETERS OF CURCUMIN–LECITHIN–PIPERINE AND BCM-95^®^CG (BIOCURCUMAX™)

Parameter	Curcumin- lecithin- piperine	BCM-95^®^CG (Biocurcumax™)
Tmax	3.5	4.67
Cmax	344.32	689.18
Ke	0.3372	0.139
t 1/2	2.245	5.323
AUC (0 - inf)	624.26	3975
Cl (observed)/F	5.715	0.624
Vz (observed)/F	22.117	4.790

This table shows the average value of pharmacokinetic parameters of curcumin-lecithin-piperine and BCM-95^®^ CG (Biocurcumax™). Tmax: Time of Peak plasma concentration, Cmax: Peak plasma concentration, Ke: Elimination rate constant, t1/2: Half life, AUC(0-infinity): Area under curve from ‘0’ h to infinity, Cl/F: Clearance/Bioavailability, Vd/F: Volume of distribution/bioavailability

Results of the present pilot study indicate that curcumin is absorbed early and retained longer from the BCM-95^®^CG (Biocurcumax™) composition compared to normal curcumin as well as from curcumin-lecithin-piperine combination. The results also indicate a probable role for the non-curcuminoid components of turmeric (especially Ar-turmerone) in the absorbability of curcumin *in vivo*.
